# Early substance use disorders and subsequent NEET-not in education, employment or training—a national cohort study

**DOI:** 10.1093/eurpub/ckad105

**Published:** 2023-06-29

**Authors:** Hélio Manhica, Diego Yacamán-Méndez, Hugo Sjöqvist, Andreas Lundin, Anna-Karin Danielsson

**Affiliations:** Department of Global Public Health, Karolinska Institutet, Stockholm, Sweden; Department of Global Public Health, Karolinska Institutet, Stockholm, Sweden; Centre for Epidemiology and Community Medicine, Stockholm, Sweden; Department of Global Public Health, Karolinska Institutet, Stockholm, Sweden; Department of Global Public Health, Karolinska Institutet, Stockholm, Sweden; Centre for Epidemiology and Community Medicine, Stockholm, Sweden; Department of Global Public Health, Karolinska Institutet, Stockholm, Sweden

## Abstract

**Background:**

Substance use problems have been associated with poor labour market outcomes. This study investigated whether substance use disorders (SUD) in emerging adulthood increase the likelihood of later being not in employment, education or training (NEET).

**Methods:**

A national cohort study of 23 5295 males and 227 792 females born between 1981 and 1987. SUD was assessed between ages 17 and 24 years. Logistic regression models were used to estimate the odds ratios (ORs) of NEET, between ages 25–34. Sibling-comparison analysis was performed to account for potential shared genetic and environmental factors.

**Results:**

Having been diagnosed with a SUD was associated with the likelihood of being NEET among males [OR = 1.37, 95% confidence interval (CI), 1.25–1.49] and females (1.19, 1.13–1.27) after adjusting for domicile, origin, psychiatric diagnosis and parental psychiatric diagnosis. Early SUD was also associated with a gradual increase in the ORs of accumulation of years being NEET. This was more evident among females. In the sibling-comparison analysis, we found a higher OR of NEET among same-sex sibling males 1.39 (1.06–1.82) and females 1.28 (0.99–1.66) with SUD. These risks were fully attenuated when another psychiatric diagnosis was adjusted for.

**Conclusion:**

Early SUD was associated with an increased likelihood of being NEET in both males and females. Neither origin, domicile, psychiatric diagnoses nor parental psychiatric diagnoses did fully explain the association. The combination of unmeasured familial factors and having other psychiatric disorders largely explained these associations.

## Introduction

Youths across the Organization for Economic Cooperation and Development (OECD) countries are confronted with diverse challenges in transiting from education to working life. Compared with the general adult population, youths are more likely to face periods of unemployment, job insecurity, temporary working contracts and other low-paid types of employment.^1^ Scholars have used the concept of not in employment, education or training (NEET) as an indicator for capturing and understanding youths’ multifaceted difficulties in the labour market and educational system. This indicator also incorporates all youths who suffer from long-term sickness or are otherwise unable to work or not available for work.^2^^,^^3^ The prevalence of NEET across the OECD countries varies substantially between age groups. For example, in 2017, the proportion of NEET was 6% among those between 15 and 19 years old, compared with 16% among those between 20 and 24, and 18% between 25 and 29-year-olds.^4^ Also, prior studies in Sweden show men to be at higher risk of being NEET compared with women.^5^

Youths who are NEET are often considered to be more vulnerable than their peers, as they are at increased risk of poorer social and health outcomes later in life.^6–9^ Therefore, identifying the determinants and underlying mechanisms leading to NEET might help to design adequate policy responses to improve employment and social integration, and also prevent lasting public health consequences. Several studies have identified different risk factors associated with being NEET; for instance, having mental health problems,^10^^,^^11^ frequent use of cannabis,^11^ growing up in poor socioeconomic environments,^12^ migrant background^13^ and having parents with substance use disorders (SUDs).^14^^,^^15^ However, the influence of early SUDs on the likelihood of later being NEET remains unknown. In addition, to our knowledge, there are no studies investigating whether being NEET following earlier SUD is a persistent or a temporary phenomenon.

Shared genetic and familial factors have been associated with psychiatric disorders, including SUDs.^16^^,^^17^ Existing studies on the risk factors associated with being NEET are limited due to the inability to account for such factors, making it difficult to establish potential causal relationships. Moreover, familial factors such as growing up in poverty, parental and siblings’ substance use and use disorders, lack of parental supervision and monitoring and poor mental health, have been found to predict substance use and subsequent disorders.^18–21^ Being NEET has also been associated with parental SUDs and parental socioeconomic conditions.^14^^,^^15^

Substance use is most common during emerging adulthood (covering the ages ∼18 to ∼25).^22^ For instance, in 2018, the prevalence of binge drinkers and drug users across the USA in the past month was highest among emerging adults compared with those aged 26 or older.^23^ Similarly, cannabis use in Canada has been found to be higher among emerging adults than in any other age group.^24^ In Sweden, the prevalence of alcohol dependence has been reported to be higher among 19–25-year-olds compared with older adults.^25^ Emerging adulthood is not only an important transition period in life characterized by important changes in lifestyle, but also a period of increased independence and usually, the introduction of individuals into the labour market.^22^^,^^26^ Therefore, exposure to SUDs during this period of life might have long-lasting effects on individuals’ social, economic and health outcomes.

In this study, we aimed to explore the associations between SUD in emerging adulthood, and the likelihood of later being NEET among males and females, separately.

Specifically, we wanted to answer the following questions:

1. What is the likelihood of being NEET in adults who were diagnosed with SUDs in emerging adulthood, relative to those who were not?
2. Is being NEET following earlier SUD a persistent or a temporary phenomenon in adulthood?
3. To what extent is being NEET following earlier SUD explained by sex, origin, domicile, another psychiatric diagnosis and parental psychiatric diagnosis?
4. To what extent is being NEET following earlier SUD explained by unmeasured shared familial confounding factors?

## Methods

### Study population

The study population comprised all individuals (*n* = 463 087) born between 1981 and 1987, alive and residing in Sweden between January 1998 and December 2005, with parents registered in Sweden. Individuals who died (*n* = 7806) and those who had at least one year of being NEET before their 25th birthday (*n* = 272 604) were excluded from the analyses. This study adhered to the Reporting of Observational Studies in Epidemiology (STROBE) Statement (Supplementary table S1). This study was done without patient or public involvement.

### Exposure variable

For this study, SUD in emerging adulthood was defined as the first diagnosis of SUD between the ages of 17 and 24 years. Diagnosis of SUD was obtained from the Swedish National in- and out-patient registers, held by the Swedish National Board of Health and Welfare, using the following codes of the tenth edition of the World Health Organization International Classification of Disorders (ICD-10): alcohol-related disorders (F10), opioids (F11), cannabinoids (F12), sedatives, hypnotics, anxiolytics (F13), cocaine (F14), other stimulant-related disorders (F15), hallucinogens (F16), volatile solvents (F18) and other psychoactive substance-related disorders and unspecified psychoactive substance-induced disorders (F19).

### Outcome variable

The definition of NEET applied in our study was based on a model created by the European Statistical Office (Eurostat) for estimating the prevalence of youths who have remained outside education, employment or training for 6 months or more during the preceding 12 months.^2^^,^^27^ The occurrence of NEET was measured when the cohort members were between 25 and 34 years of age, between 2006 and 2016. The definition of NEET was based on data on income sources according to the longitudinal integration database for health insurance and labour market studies (LISA) and the Swedish Agency for Youth and Civil Society. NEET was defined as individuals who were living and registered in Sweden for an entire calendar year, with an annual income below the price-based amount (PBA—a national statistic calculated annually from the consumer–price index), who are not receiving study loans or grants and not registered for education for more than 60 h.^28^ In addition, the definition of NEET used in this study would include women and men taking parental leave as employed given the regulations for parental leave in Sweden. Besides, NEET included those incomes from social insurance that are linked to employment, such as sickness allowances and payments from parental leave.

In our study, NEET was assessed annually as a binary variable with the value of 1 (being NEET) or 0 (no NEET) each year between January 2006 and December 2016.

### Covariates

The following covariates were assessed at baseline: *Origin* was categorized as: (i) *Native Swedish*, when born in Sweden with both parents born in Sweden; (ii) *Offspring of migrants*, who were all Swedish born with at least one parent born abroad; and (iii) *Youth migrants*, comprised individuals born outside Sweden with both parents also being born abroad, according to the Multi-Generation Register. *Domicile* of residence was categorized as: *Big city—*referred to the metropolitan areas of Sweden’s three largest cities: Stockholm, Gothenburg and Malmö. *Medium-sized town—*covered other predominately urban municipalities and *rural areas—*the remainder. This categorization was in concordance with the Swedish Association of Local Authorities and Regions.^29^  *Psychiatric diagnosis*: referred to the history of any psychiatric disorder (excluding SUDs) captured from birth until the age of 24 years, according to the Swedish National in and out-patient registers. *Parental psychiatric diagnosis* referred to the parental history of hospital care with any psychiatric diagnosis, including SUDs from the child’s birth up to 24 years of age. Sibling analysis was conditioned in same-sex siblings with at least one episode of NEET during the study period, individuals were required to have the same biological mother and father, which resulted in a total of 39 287 individuals available for this subanalysis. There were 4556 cases (1%) of missing values linked to domicile that were removed from the main analyses.

### Statistical analyses

First, we analysed the socio-demographic characteristics of the study population stratified by sex. Second, we calculated the associations between having a diagnosis of SUD in emerging adulthood (17–24 years of age) and subsequent being NEET for males and females, separately using logistic regression models. Results were presented in three different models, as odds ratios (ORs). In all models, those with no SUD during emerging adulthood were chosen as the reference category. Model 1 was the unadjusted model. Model 2 was adjusted for domicile, origin and parental psychiatric diagnosis. Model 3 adjusted for all aforementioned variables and other psychiatric diagnosis.

Third, we tested the associations between SUD and the accumulation of years in NEET as a secondary outcome. For males and females, we created indicator variables of those with 1, 2, 3 and 4 or more years in NEET during adulthood. We estimated the ORs for each of these groups compared with never being in NEET using logistic regression models. Results were presented using the three models mentioned above.

Fourth, we conducted sibling analyses using within-model stratification, thus accounting for unmeasured familial confounding factors. Logistic regression models were used to estimate the ORs of being NEET, conditioning on siblings in families of same-sex siblings with at least one episode of NEET. Results were presented in two models as ORs with 95% CIs. Model 1 was unadjusted and Model 2 was adjusted for psychiatric diagnosis. The sibling analyses focused on same-sex siblings, as the main analyses were stratified by sex.

In the sensitivity analyses, we conducted interaction analyses using the likelihood-ratio (LR) test of exposure to SUD and origin, parental psychiatric diagnosis and psychiatric diagnosis on the odds of being NEET. The LR test involves estimating two models and comparing them (the model without the interaction term and with the interaction term). Based on the statistical significance of the LR test, we stratified the analyses. We also conducted additional logistic regression analyses on siblings to assess for possible potential selection bias in the sibling sample. We compared the incidence of NEET from ages 25 to 34 among males and females with an earlier diagnosis of SUD. Further, we calculated OR of NEET among males and females, separately after adjusting for origin, domicile, parental psychiatric condition, psychiatric condition and the number of children. The variable ‘number of children’ referred to the number of children aged 0–6 years in the household, that is, who are registered at the same residential address as the study participants.

## Results

Among the 463 087 individuals included in the main analyses (table 1), around 13% of males and 43% of females had at least one episode of NEET between the ages of 25 and 34 years. Among males who experienced being NEET, about 2.7% had a previous SUD. The majority of men were native Swedish (72%) and lived in medium-sized towns (45%). About 12% of them had at least one parent with a psychiatric diagnosis, 7% had a psychiatric diagnosis and the majority (90%) were childless when they were 24 years of age.

**Table 1:** Socio-demographic characteristics and rates of being NEET by covariates among males and females aged 25 and 34 years

Variables	Males		Female	
	At least one year of being NEET (*n* = 31 963)	No episode of being NEET (*n* = 203 332)	At least one year of being NEET (*n* = 77 850)	No episode of being NEET (*n* = 149 942)
	*N* (%)	*N* (%)	*N* (%)	*N* (%)
SUD				
No SUD in emerging adulthood	31 102 (97.3)	147 953 (98.7)	76 432 (98.2)	200 611 (98.6)
SUD in emerging adulthood	861 (2.7)	1989 (1.3)	1418 (1.8)	2721 (1.4)
Origin				
Native Swedish	23 152 (72.4)	123 179 (82.2)	61 409 (78.9)	168 607 (82.9)
Youth migrant	3166 (6.9)	6801 (4.5)	5108 (6.6)	9400 (4.6)
Offspring of migrants	5645 (17.7)	19 962 (13.3)	11 333 (14.6)	25 325 (12.5)
Domicile				
Large city	11 886 (37.9)	58 228 (39.3)	27 177 (35.3)	66 716 (33.1)
Medium-sized town	14 525 (45.4)	71 236 (48.1)	37 214 (48.3)	101 150 (50.1)
Rural area	4948 (15.8)	18 758 (12.6)	12 680 (14.4)	34 003 (16.8)
Parental psychiatric diagnosis				
No	28 113 (87.0)	136 392 (91.0)	69 438 (89.2)	184 102 (90.5)
Yes	3850 (12.0)	13 550 (9.0)	8412 (10.8)	19 230 (9.5)
Psychiatric diagnosis				
No	29 554 (92.5)	139 965 (93.3)	71 122 (91.4)	195 486 (96.1)
Yes	2409 (7.5)	9977 (6.7)	6728 (8.6)	7846 (3.9)
Number of children				
0 children	29 813 (94.3)	135 912 (91.8)	61 141 (78.9)	182 946 (90.8)
1 children	1274 (4.0)	9744 (6.6)	13 924 (18.0)	13 821 (6.9)
2 children	474 (1.5)	2297 (1.6)	2238 (3.0)	4453 (2.2)
3 or more children	57 (0.2)	94 (0.06)	106 (0.1)	356 (0.2)

SUD = Hospital admission due to any SUD. *N* = total cases.

In the female population with at least one episode of being NEET, about 1.8% had a prior SUD diagnosis. The majority were native Swedish (79%) and lived in medium-sized towns (48%). Nearly 10.8% of the females had a psychiatric diagnosis and approximately 8% of them had at least one parent with a psychiatric diagnosis. About 20% of females being NEET had at least one child before the age of 25.

Being diagnosed with SUD at ages 17–24 years was associated with increased likelihood of being NEET between ages 25 and 34 years (table 2), OR = 2.00 (1.85–2.16, 95% CI) in males and 1.37 (1.28–1.47) in females when adjusting for domicile, origin and parental psychiatric diagnosis. In the fully adjusted model, the ORs were further attenuated to 1.37 (1.25–1.49) and 1.19 (1.13–1.27), for males and females, respectively.

**Table 2:** Logistic regression models of NEET by SUD in emerging adulthood in males and females, separately between 25 and 34 years of age

Exposure	OR (95% CI)	OR (95% CI)	OR (95% CI)
Males	Model 1	Model 2	Model 3
No SUD in emerging adulthood	Ref	Ref	Ref
Had SUD in emerging adulthood	2.04 (1.89–2.20)	2.00 (1.85–2.6)	1.37(1.25–1.49)
Females			
No SUD in emerging adulthood	Ref	Ref	Ref
Had SUD in emerging adulthood	1.38 (1.28–1.47)	1.37 (1.28–1.47)	1.19 (1.13–1.27)

*Notes*: Model 1 unadjusted. Model 2 adjusted for country of origin, domicile and parental psychiatric diagnosis. Model 3 additionally adjusted for psychiatric diagnosis (fully adjusted model). CI = confidence interval; OR = odds ratio; Ref = reference category; *N* = population; SUD = hospital admission due to SUD.

**Figure 1:**
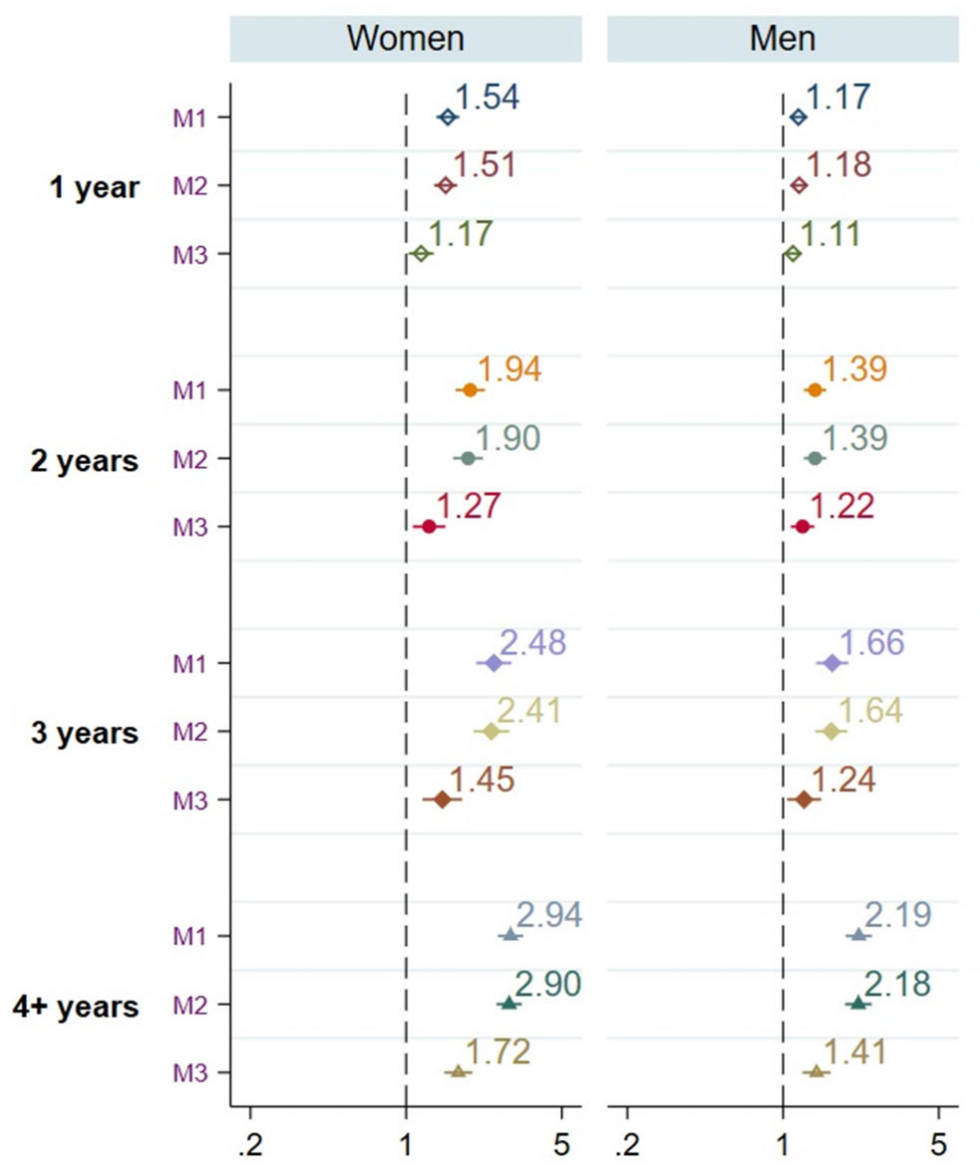
Logistic regression models of the association between SUD in emerging adulthood on the accumulation of years being NEET for -female (*n* = 227 792) and males (*n* = 235 295), separately aged 25 and 34 years. M1 = Model 1 (unadjusted); M2 = Model 2 (adjusted for country of origin, domicile and parental psychiatric diagnosis); M3 = Model 3 (additionally adjusted for psychiatric diagnosis)

Females with a previous SUD diagnosis had a gradual increase in the odds of accumulation of years of being NEET, relative to those who never experienced NEET (figure 1). In the fully adjusted model, the ORs for being NEET 1 year were 1.17 (95% CI, 1.02–1.32); 2 years, 1.27 (1.07–1.50); 3 years, 1.45 (1.18–1.78) and more than 4 years 1.72 (1.48–1.99), respectively. For males, the corresponding ORs were 1.11 (1.01–1.21); 2 years, 1.22 (1.08–1.38); 3 years, 1.24 (1.04–1.48) and more than 4 years, 1.41 (1.22–1.63).

When comparing the ORs of NEET between same-sex siblings being discordant in NEET status (table 3), the ORs of being NEET after being diagnosed with a SUD in males were OR = 1.39 (95% CI 1.06–1.82) and in females 1.28 (1.00–1.66). No associations were found in males 0.10 (0.82–1.48) and females 1.16 (0.88–1.52), when we adjusted for other psychiatric diagnoses.

**Table 3:** Logistic regression models for NEET by SUDs in emerging adulthood in males and females aged 25 and 34 years of age—conditioning on same-sex siblings with at least one episode of NEET

Exposure	*N*	NEET	OR (95% CI)	OR (95% CI)
Males			Model 1	Model 2
No SUD in emerging adulthood	9765	4614	Ref	Ref
Had SUD in emerging adulthood	215	123	1.39 (1.06–1.82)	1.10 (0.82–1.48)
Females				
No SUD in emerging adulthood	18 311	9087		
Had SUD in emerging adulthood	240	133	1.28 (0.99–1.66)	1.16 (0.88–1.52)

Model 1 unadjusted model; Model 2 adjusted for psychiatric diagnosis. CI = confidence interval; OR = odds ratio; Ref = reference category. *N* = sibling pairs. SUD = hospital admission due to SUD.

In the sensitivity analyses, we found a statistically significant interaction effect of origin (Supplementary table S2) and psychiatric diagnosis (Supplementary table 3) on the odds of being NEET. The performed LR tests are presented as Supplementary Material (Supplement A). Analyses stratified by origin suggested that the ORs of NEET were higher among native Swedish males and migrant offspring with SUD, after adjusting for domicile, parental psychiatric diagnoses and psychiatric diagnoses. In females, the OR was higher among native Swedes and migrant offspring with SUD in emerging adulthood. When we also tested the role of SUD on the likelihood of NEET stratified by psychiatric diagnosis; results suggested that the effect of SUD on the odds of being NEET was higher among males without a psychiatric diagnosis.

Furthermore, to explore the possibility of selection bias in the sibling sample used for the main analysis, we repeated the analysis using a sibling sample without conditioning the analyses to same-sex siblings nor those with at least one episode of NEET (Supplementary table S4), the results of the association between SUD in emerging adulthood and NEET in adulthood were approximately equal to those of the general models, suggesting that there was no bias in the siblings’ sample.

We compared the incidence of NEET from ages 25 to 34 among males and females with early diagnosis of SUD. Regardless of early SUD, the incidence of NEET (Supplementary figure S1) was higher in females than in males. Additionally, we also tested the associations between being diagnosed with SUD and the likelihood of accumulation of years in NEET, for males and females, separately, after adjusting for all aforementioned variables and the number of children in the household at the age of 24 (Supplementary table S4). Results for males and females were quite similar to those reported in the fully adjusted model 3, reported in table 2.

## Discussion

This study showed that being diagnosed with SUD in emerging adulthood was associated with an increased likelihood of being NEET for both males and females, after adjusting for origin, domicile and parental psychiatric diagnosis. This association was partially, but not fully, attenuated after adjusting for own psychiatric diagnosis. Our results also showed a gradual increase in the accumulation of years of being NEET among those diagnosed with SUD, which was most pronounced among females. Additional analyses suggested the association between early SUD and NEET was largely explained by the combined effect of shared familial factors and individual psychiatric conditions.

Our findings are in line with other studies showing that early mental health problems and frequency of cannabis use or alcohol use are associated with being NEET, job loss or poor educational attainment.^11^^,^^30–32^ Our additional findings (Supplementary table S3) that being exposed to earlier SUD and the odds of being NEET seemed to be higher among males without a psychiatric diagnosis, might suggest that some of the effect might overlap between psychiatric diagnoses and SUD status in relation to NEET status. This pattern would not necessarily mean that those with a psychiatric diagnosis show more resilience, but rather evidence of notable correlation between SUD and non-SUD psychiatric outcomes.

Our results also showed a marginal influence of having children before the age of 25 and a later likelihood of being NEET. This could partly be explained by childbearing being relatively uncommon among younger males and females in Sweden. In addition, our findings showed that the incidence of being NEET between the ages of 24 and 34 after having been diagnosed with SUD in emerging adulthood was generally higher among females than males. Factors associated with family responsibilities, such as motherhood and the provision of childcare^33^ might play a role in explaining why these females might face disadvantages with regards to labour market participation with consequent wage disadvantage compared with men. Our additional findings (Supplementary figure S2) are consistent with those reported by the Labor Force Surveys and the Living Conditions,^34^ highlight the importance of childbearing for the existence of gender gap in NEET. In fact, a large part of the NEET sex gap among those aged 25 and 34 years can be explained by an increased prevalence of women but not men, with long part-time employment; about 30% and 10%, respectively, affecting the total earned income in 2017.

Whereas males exposed to early SUD seemed to have higher odds of later being NEET, the gradual increase in accumulation of years of being NEET was more pronounced among females. In addition, our study indicated that females with SUD are more likely to accumulate years being NEET than their male counterparts and that they also had two times higher probability of being NEET between the ages of 25 and 34, even if they did not have an early diagnosis of SUD.

Youths with a migrant background in Sweden are over-represented among those being NEET.^12^^,^^13^ Still, we found no associations between SUD and being NEET in the migrant population. This is partly explained by the lower rates of SUDs in migrants compared with the Swedish-born population,^35^ which in turn may reflect a lower relatively lower utilization of mental health services among migrant youth,^36^ as well as a risk of underdiagnosis of mental health conditions.^37^

Our general findings suggest that the potential confounders available in the register data (e.g. domicile, origin, other psychiatric diagnoses and parental psychiatric diagnoses) explained a rather substantial part of the associations between early SUD and later NEET. We used sibling comparison models to account for non-measured confounding factors shared between siblings. Results suggested that familial factors—for example, genetic variation or shared familial environmental risk factors for early SUD and NEET were quite similar for males and females. In addition, when the psychiatric diagnoses were added to the sibling comparison models, the results suggested that the association between early SUD and NEET was largely explained by the combined effect of familial factors and individual psychiatric conditions.

## Strengths and limitations

A major strength of this register-based study was that it was based on data from a combination of national registers covering the entire population living in Sweden. The use of these registers allowed us to analyse the study population longitudinally, females and males separately and adjust for important confounding variables. In addition, we conducted sibling analyses to account for potential unmeasured familial confounding factors.

This study also has some limitations. For example, being NEET and having a SUD might share many additional overlapping risk factors (e.g. family situation, health status and neighbourhood), on which we had no information. Still, the sibling analyses might resolve some of those issues.

The variables of SUDs and other psychiatric disorders have some weaknesses, which may impact the results. Importantly, we used primary diagnosis only to identify individuals with SUD from national registries. Additionally, since diagnosis are based on those who have been in contact with specialized health care and received a diagnosis, this also reflects healthcare-seeking behaviours. Individuals who face barriers to seeking care for substance use problems and mental health problems are not captured in the registers. As a result, the true proportion of individuals with SUD and mental health problems is likely to be higher than those reported in our study. However, those classified as having SUD according to a primary diagnosis are less likely to be an incorrect diagnosis. Bias due to misclassification of exposed individuals as unexposed would lead to a dilution of the effect towards the null, we can therefore be reasonably confident that the effects reported in this study are likely to be an underestimation of the true effect.

The variable NEET also has some weaknesses that should be considered when interpreting the results. NEET correspond to a heterogeneous population group,^2^ with very different experiences, characteristics and needs, encompassing both vulnerable and non-vulnerable individuals.^38^ Additionally, the definition of NEET used in this study was derived from data on income sources and education. Consequently, individuals with unknown activity or undeclared income and those who were travelling or working abroad (but were still registered in Sweden) may have been defined as being NEET.

Sibling analyses are used to account for shared familial confounding—both environmental and genetic—but should be interpreted with caution, due to their tendency to bias the risk towards the null.^39^ Another limitation of sibling analyses is a risk of selection bias, a result of restricting the sample to families with same-sex siblings, wherein at least one has a diagnosis of SUD.

## Conclusion

Our study showed that being diagnosed with a SUD in emerging adulthood was associated with an increased likelihood of later being NEET for both males and females. Neither origin, psychiatric diagnoses nor parental psychiatric diagnoses did fully explain this association. However, the sibling analysis suggested that familial factors—for example genetic variation or shared environment and having another psychiatric diagnosis—largely explained the association between an early SUD and later being NEET.

## Ethical approval

The Regional Ethics Committee in Stockholm approved the study before any records were linked (decision number: 2010-1185-31-5).

## Supplementary Material

ckad105_Supplementary_Data

## Data Availability

The dataset analysed during the current study are not publicly available owing to the Swedish data protection laws that restrict public sharing of data. However, we are happy to answer any questions about the data used in this study and to share the statistical codes and unpublished results. The Swedish national registers are protected by special legislation that makes it possible for researchers to collect certain information without personal consent. The dataset used in this study is based on multiple linked data from national routine registers. The dataset is anonymous and the researchers have no access to any personal information that could identify individuals included in the dataset. Key points Males and females who have been diagnosed with SUD in emerging adulthood have a greater likelihood of being NEET between ages 25 and 34 years. Females diagnosed with SUD were more likely to accumulate years of being NEET than males. Public health policies aiming at reducing inequalities in labour market participation are needed. Such policies should address the role of familial factors and individual mental health status.
